# Target enrichment sequencing of 307 germplasm accessions identified ancestry of ancient and modern hybrids and signatures of adaptation and selection in sugarcane (*Saccharum* spp.), a ‘sweet’ crop with ‘bitter’ genomes

**DOI:** 10.1111/pbi.12992

**Published:** 2018-08-29

**Authors:** Xiping Yang, Jian Song, James Todd, Ze Peng, Dev Paudel, Ziliang Luo, Xiaokai Ma, Qian You, Erik Hanson, Zifan Zhao, Yang Zhao, Jisen Zhang, Ray Ming, Jianping Wang

**Affiliations:** ^1^ Agronomy Department University of Florida Gainesville FL USA; ^2^ Sugarcane Research Unit USDA‐ARS Houma LA USA; ^3^ Center for Genomics and Biotechnology Key Laboratory of Genetics, Breeding and Multiple Utilization of Corps Ministry of Education Fujian Provincial Key Laboratory of Haixia Applied Plant Systems Biology Fujian Agriculture and Forestry University Fuzhou Fujian China; ^4^ Department of Plant Biology University of Illinois at Urbana‐Champaign Urbana IL USA

**Keywords:** *Saccharum* spp., sugarcane, germplasm, target enrichment sequencing, linkage disequilibrium, selective sweeps, domestication, selection, environmental association analysis

## Abstract

Sugarcane (*Saccharum* spp.) is a highly energy‐efficient crop primarily for sugar and bio‐ethanol production. Sugarcane genetics and cultivar improvement have been extremely challenging largely due to its complex genomes with high polyploidy levels. In this study, we deeply sequenced the coding regions of 307 sugarcane germplasm accessions. Nearly five million sequence variations were catalogued. The average of 98× sequence depth enabled different allele dosages of sequence variation to be differentiated in this polyploid collection. With selected high‐quality genome‐wide SNPs, we performed population genomic studies and environmental association analysis. Results illustrated that the ancient sugarcane hybrids, *S. barberi* and *S. sinense*, and modern sugarcane hybrids are significantly different in terms of genomic compositions, hybridization processes and their potential ancestry contributors. Linkage disequilibrium (LD) analysis showed a large extent of LD in sugarcane, with 962.4 Kbp, 2739.2 Kbp and 3573.6 Kbp for *S*. *spontaneum*,* S*. *officinarum* and modern *S*. hybrids respectively. Candidate selective sweep regions and genes were identified during domestication and historical selection processes of sugarcane in addition to genes associated with environmental variables at the original locations of the collection. This research provided an extensive amount of genomic resources for sugarcane community and the in‐depth population genomic analyses shed light on the breeding and evolution history of sugarcane, a highly polyploid species.

## Introduction

Sugarcane (*Saccharum* spp.) is an important cash crop extensively used for sugar production and increasingly used for bio‐ethanol production (Dahlquist, 2013). It contributes up to 80% of sugar in the world and provides ~60% of bio‐fuel globally. As a perennial C_4_ grass, sugarcane is primarily grown in the tropical and subtropical regions in more than 100 countries on ~27.1 million hectares (FAO, 2014). Sugarcane has been cultivated for more than 2000 years in India and China, the top countries for current sugarcane production (Henry, 2010; Mukherjee, 1957).

Sugarcane belongs to the genus *Saccharum*, which was considered to consist of six species, two wild species *S. spontaneum* and *S. robustum*, and four cultivated species *S. officinarum*,* S. barberi*,* S. sinence* and *S. edule* (Daniels and Roach, 1987). Recent research classified the genus *Saccharum* into only two species, *S*. *officinarum* (2*n* = 80, *x* = 10) and *S*. *spontaneum* (2*n* = 40–128, *x* = 8) (Irvine, 1999; Nayak *et al*., 2014). The domesticated species *S. officinarum* is the major genome contributor to modern sugarcane cultivars (D'Hont, 2005), and widely cultivated for its ability to accumulate sucrose but vulnerable to several diseases. Meanwhile, *S. spontaneum* is a widely adapted wild species that is resistant to disease and stress. Therefore, in 1885, Soltwedel firstly initiated an inter‐specific breeding programme to cross *S. officinarum* (Loethers) with *S. spontaneum* (Glagah) to develop plants resistant to sugarcane sareh disease virus (Bremer, 1961). Since then the inter‐specific hybrid cultivars were developed and back crossing between these hybrids and *S*. *officinarum* lines continued and led to some successful varieties, such as 2722 P.O.J., 2725 P.O.J., 2875 P.O.J., 2878 P.O.J. and some Co clones in India, which were found in sugarcane pedigrees worldwide (Breaux and Koike, 1978; Deren, 1995; Shrivastava and Srivastava, 2016). Thus, the modern sugarcane cultivars are complex hybrids derived from inter‐specific hybridization, mostly between *S. officinarum* and *S. spontaneum*.

In order to meet the increasing world demand for sugar and renewable energy, sugarcane production and stress tolerance need to be improved, which has been an enormous task with significant challenges. One of challenges is the genome complexity, which are all highly polyploidy or even be aneuploid. Modern sugarcane cultivars have a chromosome number of 100–130 with approximately 80% chromosomes from *S. officinarum,* 10% chromosomes from *S. spontaneum* and 10% recombinant chromosomes (D'Hont *et al*., 1996), and an estimated genome size of approximately 10 Gbp (D'Hont, 2005). Thus, the “bitter” complex genome makes sugarcane genetic and genomic studies highly daunting tasks. Even with the advance of next‐generation sequencing (NGS) technologies being widely applied for whole‐genome sequencing (WGS) and re‐sequencing of many crops, the reference genome for sugarcane is still not publicly available yet. While WGS is more comprehensive, it is still prohibitive to sequence complex genomes in their entirety due to high cost, time and resource requirements. Therefore, ‘target‐enrichment’ methods that selectively capture genomic regions from a DNA sample before sequencing are gaining popularity. In sugarcane, the target enrichment method enhanced the target regions by 10–11 times greater in the sequence depth than the WGS approach (Bundock *et al*., 2012). A pilot experiment has proven that target enrichment sequencing could effectively capture genomic regions of interest in sugarcane to detect allelic sequence variations (Song *et al*., 2016). Another challenge of the cultivar improvement is the narrow genetic resources in the breeding programmes with only handful germplasm contributors to the current cultivars (Deren, 1995), although sugarcane has over hundred years of breeding history. Maintenance, characterization and utilization of genetic diversity in sugarcane are critical for breeding superior cultivars with improved sugar yield and stress tolerance. The large diverse sugarcane germplasm collection, especially the “world collection of sugarcane and related grasses” (WCSRG) assembling a significant amount of genetic resources (Nayak *et al*., 2014; Todd *et al*., 2014). A core germplasm collection was formed by selecting representative accessions from the WCSRG (Nayak *et al*., 2014; Todd *et al*., 2014), which showed significant amount of natural phenotypic variations, and is essential for sustained sugarcane cultivar improvement.

To obtain a global overview of sequence variations and a refined population structure of sugarcane germplasm, and to illustrate the ancestry contributions and footprints of domestication and selection of sugarcane, we deeply sequenced the coding regions of 307 accessions selected from the WCSRG (Nayak *et al*., 2014) by using target‐enrichment sequencing. Large number of sequence variations was called with high‐allele dosage resolution. Genome‐wide single‐nucleotide polymorphisms (SNPs) were used to evaluate the genetic relationships of the collection. Mislabelled or unknown accessions were commented for new assignment. The genome compositions of ancient sugarcane hybrids, *S. barberi* and *S. sinense*, and modern sugarcane hybrids were calculated, highlighting a strong recurrent selection in modern *S. *hybrids in breeding. Large extent of linkage disequilibrium (LD) was firstly revealed for the sugarcane progenitor species and hybrids. We discovered 75 selective sweeps and 1723 candidate genes under domestication and selection controlling important agronomic traits. We also identified 121 SNPs landing in 106 genes significantly associated with environmental variables. This research not only provided valuable genomic resources for sugarcane community but also closely overviewed the germplasm population structures and genomic signatures of the crop in breeding and evolution history, which will shed light on genomic research of polyploid species with complex genomes.

## Results

### Target enrichment sequencing and novel variants identification

**Figure 1:**
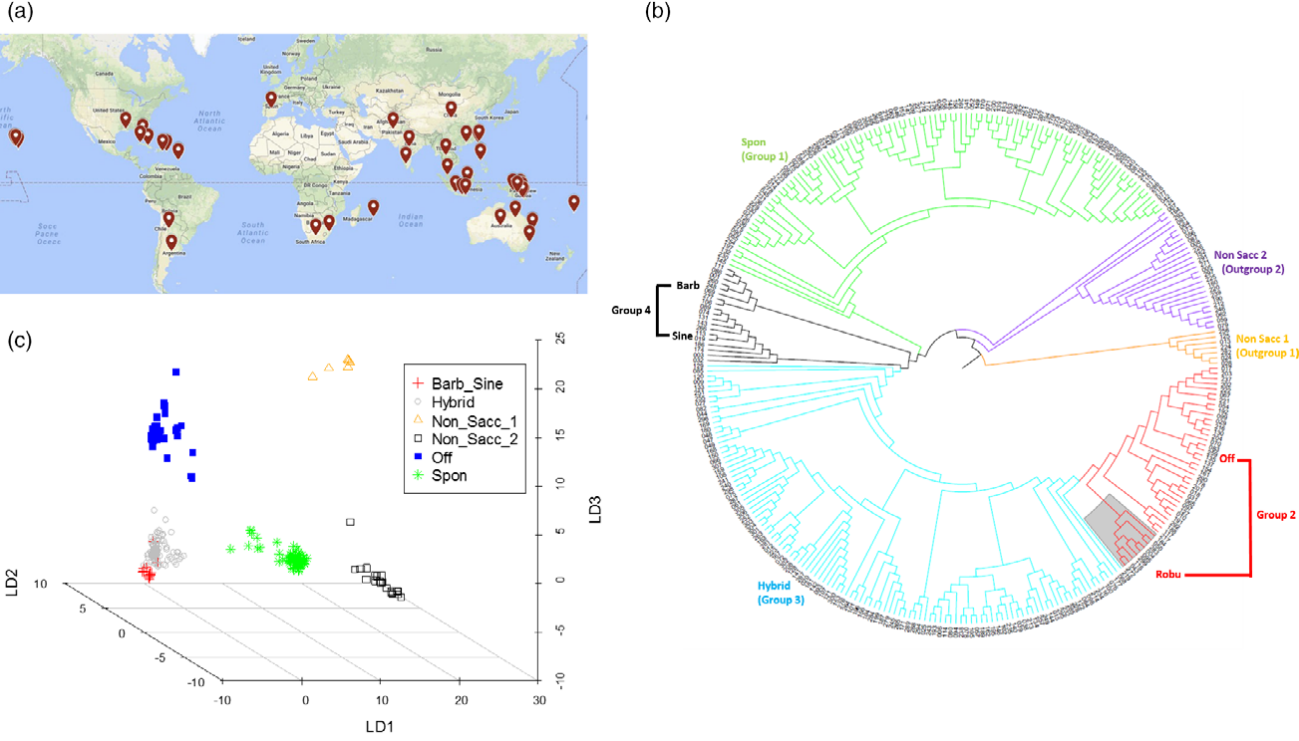
An overview of the diversity of the sugarcane accessions. (a) Geographical distribution of the 299 accessions selected from the World Collection of Sugarcane and Related Grasses. (b) Phylogenetic tree of the 307 accessions. (c) Discriminant analysis of principal components analysis of the 307 sugarcane accessions. Robu = *S. robustum*; Spon = *S. spontaneum*; Off = *S. officinarum*; Hybrid = modern *S*. hybrids; Barb = *S. barberi*, Sine = *S. sinence*; Non sacc = Non *saccharum*.

**Figure 2:**
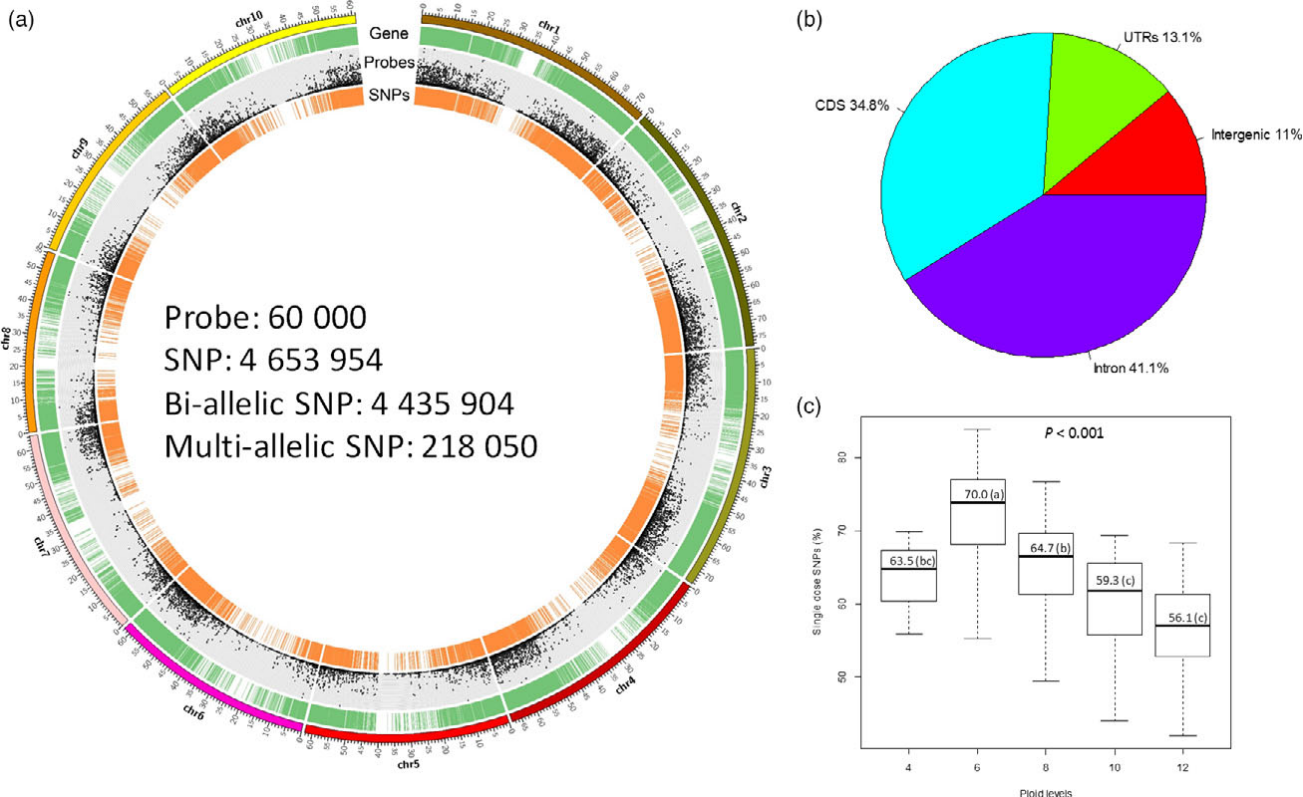
Summary of target‐enrichment sequencing data of the diversity panel. (a) Genome‐wide distribution of gene models, probes and SNPs according to sorghum genome v3.0. Gene models are displayed by green ring. Dot plot represents the number of probes. The SNP set1 is plotted in orange line. The figure was plotted within 1 Mbp window size. (b) SNP distribution according to sorghum gene models v3.0. (c) Percentages of single‐dose SNPs characterized according to ploid levels. SNP, single‐nucleotide polymorphisms; UTR, untranslated region; CDS, coding sequences.

We assembled a sugarcane diversity panel of 307 accessions including 299 accessions originally collected from 25 different countries in eight geographical regions (Oceania‐Pacific, East Asia, North America, South America, South Asia, Caribbean region, South Africa and Europe) to represent the wide genetic diversity of the WCSRG (Figures 1a, S1, and Table S1) and eight modern sugarcane clones selected from the sugarcane breeding programme in Florida, the USA. For target enrichment sequencing of this diversity panel, we designed a total of 60 thousand probes (Data S1) to enrich coding regions of the genome for sequencing. Of the designed probes, 55 588 were derived from transcript sequences corresponding to 16 846 gene models according to the sorghum genome v3.0 (Paterson *et al*., 2009). To expand the potential of even distribution of the target regions with 1 probe per 12 Kbp (Figure 2a), 4230 probes were designed from intergenic regions in the genome. The remaining 182 probes (0.3% of the total) could not be mapped to the sorghum genome as they were designed from sugarcane‐specific regions, and most of them (79.1%) were validated with high efficiency to fish out sugarcane genome in our pilot experiments (Song *et al*., 2016).

Overall, 7.9 billion clean reads were generated after sequencing the target regions of 307 accessions with 7.27 billion (92.1%) clean reads mapped to sorghum genome v3.0 and 7.10 billion (90.7%) uniquely mapped (in a range of 61.2% to 96.0% across the accessions). The mapped reads covered 65.2 Mbp (8.9%) of the sorghum genome at an average depth of 98× in a range of 9× to 375× (Table S2). After level 1 filtering, 4.65 million SNPs were identified from the uniquely mapped reads, representing a comprehensive catalogue of *Saccharum* spp SNP database with 4.43 million bi‐allelic SNPs and 218 K (4.7%) multi‐allelic SNPs. The SNP density was 163.4 SNPs/Kbp, ranging from 156.8 (chromosome 1) to 171.2 SNPs/Kbp (chromosome 7) (Figure 2a and Table S3). The number of SNPs per chromosome was correlated with the number of probes designed (Correlation coefficient = 0.9996). The SNPs were mostly located in genic regions (4.14 million, 89.0%) because the probes used to capture the targeted regions were mainly designed from gene models (Figure 2b). Of the 4.14 million genic SNPs, 13.1%, 34.8% and 41.1% were in untranslated regions (UTRs), coding sequences (CDS) and introns, respectively (Figure 2b), with an average of 150.3, 153.2 and 327.9 SNPs per Kbp respectively. Within the coding regions, 24 842 SNPs were found to have large effects, with 23 032 SNPs introducing stop codons and 1820 disrupting stop codons in 9083 and 1571 gene models respectively. Interestingly, among the genes containing at least five SNPs with large effects (1204 genes), 66 significantly enriched genes had ADP‐binding domain, compared to that of the rest of the genome [*P *<* *1e^−11^ adjusted for false discovery rate (FDR)]. Among the 66 enriched genes all of them contained nucleotide‐binding site (NBS) domain, and 20 of them contained both NBS and leucine‐rich repeat (LRR) domains. The results showed a trend of fast evolving of new alleles in putative disease resistance genes in this polyploid *Saccharum* species, which might have played a key role in the divergence of *Saccharum* from their ancestors.

After removing multi‐allelic SNPs and SNPs without reference allele (17 K, 0.4% of the *Saccharum* SNP database), 4.42 million bi‐allelic SNPs were used to calculate ratio of transition/transversion substitutions (Ti/Tv). The Ti/Tv ratio of genome‐wide SNPs was 2.28, which was lower than that of CDS (3.12) and higher than non‐CDS (2.00), in consistence with previous reports (Bainbridge *et al*., 2011; Clark *et al*., 2011; Wang *et al*., 2014). The overall Ti/Tv ratio of each chromosome was similar to that of the genome‐wide SNPs (Table S3). The overall non‐synonymous/synonymous (N/S) ratio was 0.8, which was lower than that of sorghum (1.0) (Mace *et al*., 2013), rice (1.3) (Xu *et al*., 2012) and equal to that of *Arabidopsis* (0.8) (Clark *et al*., 2007), indicating that high synonymous mutations were dominant and favoured in *Saccharum* spp. Although focusing on a subset of the genome, the general pattern of sequence variants observed here was likely similar with that of the genome‐wide sequence variants because a large number of SNPs with a high density and quality were included into the analyses.

After level 2 filtering, a total of 4.26 million SNPs, which highlighted the variations with confident dosage resolution, were characterized, and thus used for majority of subsequent data analyses requiring genotypes at each locus. With this SNP set, we calculated percentages of single‐dose (SD) SNPs among the total SNPs called for each accession. SD SNPs were predominant type comparing with SNPs of other dosages, with an average of 63.5%, 70.0%, 64.7%, 59.2% and 56.1% for tetra‐, hexa‐, octo‐, deca‐ and dodeca‐ploid species respectively (Figure 2c). The percentages of SD SNPs were significantly different for species with different ploid levels (*P *<* *0.001) and showed a decreasing trend with increased ploid levels except for tetraploid, in which only three accessions were sampled.

In addition to SNPs, 858.1 K InDels were also called with most (94.1%) of them being small (1–10 bp) in size. The majority of the InDels (81.7%) were in genic regions, with 8.1% (69 335) located in CDS, of which 49.2% led to frame‐shift mutations compared with reference alleles. In contrast, 82.7% of the InDels in non‐CDS regions were multiples of three bases in length, indicating InDels characterized were not tending to disrupt the original gene function. We also characterized 7916 PAVs and 18 991 SSRs, of which 6664 PAVs (84.1%) and 15 922 SSRs (83.8%) were in genic regions.

### Phylogenetic analysis and sugarcane sub‐populations

To determine genetic relationships of the diversity panel, we performed phylogenetic analysis among the sugarcane accessions using 156.7 K SNPs with call rates ≥98%. Four *Saccharum* groups were identified with group 1 including 101 *S. spontaneum* accessions, group 2 including 35 *S. officinarum* accessions and 9 *S. robustum* accessions, group 3 including 111 modern *S. *hybrids accessions and 19 accessions of *S. barberi* and *S. sinense* as ancient hybrids in group 4 as two separate sub‐groups (Figure 1b and Table S1). *Saccharum barberi* and *S. sinense* were cultivated in India and China, respectively, for sugar production since prehistoric times (Mukherjee, 1957), but no longer grown since the modern sugarcane cultivars were developed. Both *S. barberi* and *S. sinense* as well as modern *S. *hybrids were derived from hybridization between *S. spontaneum* and *S. officinarum*. However, accessions of *S. barberi* and *S. sinense* (Figure 1b, black colour) were clustered together, separated from modern *S. *hybrids (Figure 1b, blue colour), indicating that ancient sugarcane hybrids had unique genetic features compared to modern *S. *hybrids. In addition to the four groups of *Saccharum* spp, two non‐*Saccharum* outgroups were identified as outgroup 1 and outgroup 2 with 7 and 25 accessions respectively (Figure 1b and Table S1). Outgroup 1 had all *Miscanthus* accessions in the diversity panel, whereas outgroup 2 included accessions from several species that previously were named as *Saccharum* in the germplasm collection during curation, such as *S*. *arundinaceum*,* S*. *bengalense*,* S*. *brevibarbe*,* S*. *kanashiroi*,* S*. *procerum*,* S*. *ravennae* and *S*. *rufipilum*. Outgroup 2 was placed between outgroup 1 (*Miscanthus*) and the four groups of *Saccharum* based on phylogenetic analysis. Except for *S. barberi* (mainly from India), clustering of the accessions selected from the WCSRG was not correlated with their geographic origins, indicating that the spreading of sugarcane germplasm worldwide originated from the same source diversified before spreading.

In group 1, the *S. spontaneum* group, three previously misclassified accessions (Sample ID 58, 132 and 155 named as *S. arundinaceum*,* S. robustum* and *Sorghum arundinaceum,* respectively) were noticed and were thus corrected through the analysis in this study (Table S1). In addition, five accessions with no classification in the collection (Sample ID 36, 194, 200, 211 and 227) were placed into group 1, thus could be named as *S. spontaneum* accessions (green in Figure 1b). The ploidy levels (estimated using flow cytometry) ranged from 6× to 12× with 8× as the major type in the *S. spontaneum* group (Table S1). Accessions with different ploidy levels were not clustered distinctly according to the ploidy level, indicating that the genetic differentiation occurred earlier than the ploidy level differentiation in *S. spontaneum*, most likely the different ploidy levels were evolved through hybridizations among accessions of similar genetic background in this species. Accessions of *S. robustum* (highlighted in grey in Figure 1b) were clustered with the *S. officinarum* group (red in Figure 1b) in group 2, showing their close relationships and supporting that *S. officinarum* was most likely domesticated from *S. robustum*. In group 2, one accession (Sample ID 50 previously classified as *S*. *officinarum*) was most likely *S. robustum* and one unknown accession (Sample ID 17) could be classified as *S. officinarum*. Group 3 of modern *S. *hybrids (blue in Figure 1b) and group 4 of ancient hybrids, *S. barberi* and *S. sinense* (black in Figure 1b), were placed between *S. spontaneum* and *S. officinarum*. In group 4, three misclassified and three unknown accessions were noticed (Table S1) and thus renamed. The group 3 of modern *S*. hybrids was placed between the group 4 consisting of the ancient sugarcane hybrids and group 2 consisting of *S. officinarum*, and included 41 accessions as modern *S*. hybrids, 42 accessions previously named as *S. officinarum* (10 of them without clone names), 12 named as other species in the germplasm collection and 6 unknown species according to records in the WCSRG (blue in Figure 1b, and Table S1). Based on the current phylogenetic analysis, all these accessions in group 3 were most likely modern *S*. hybrids. Modern *S*. hybrids were derived from crosses between *S. spontaneum* and *S. officinarum*, and went through multiple backcrosses with *S. officinarum*, which made modern *S*. hybrids and *S. officinarum* not easily discriminable, therefore could have been misclassified during germplasm curation. For example, two accessions (Sample ID 78 and 232) were named as *S. officinarum* in the WCSRG, but were widely considered as modern *S*. hybrids in sugarcane community.

We further carried out population structure analyses using discriminant analysis of principal components (DAPC) and ADMIXTURE using 10 K SNPs (one SNP per 10 Kbp, call rates ≥98%). The results (*K* = 6 was chosen based on Bayesian information criteria value for DAPC and minimized cross‐validation error for ADMIXTURE following the manuals of the method) were mostly consistent with phylogenetic analysis with a few exceptions, dividing the collection into six groups including four *Saccharum* and two non‐*Saccharum* groups (Figures 1c and S2). Based on the consistent results of above analyses, we selected 40, 28 and 40 representative accessions representing the three sub‐populations of *S*. *spontaneum, S*. *officinarum* and modern *S*. hybrids, respectively, to conduct downstream population genomic analyses (Table S4).

**Figure 3:**
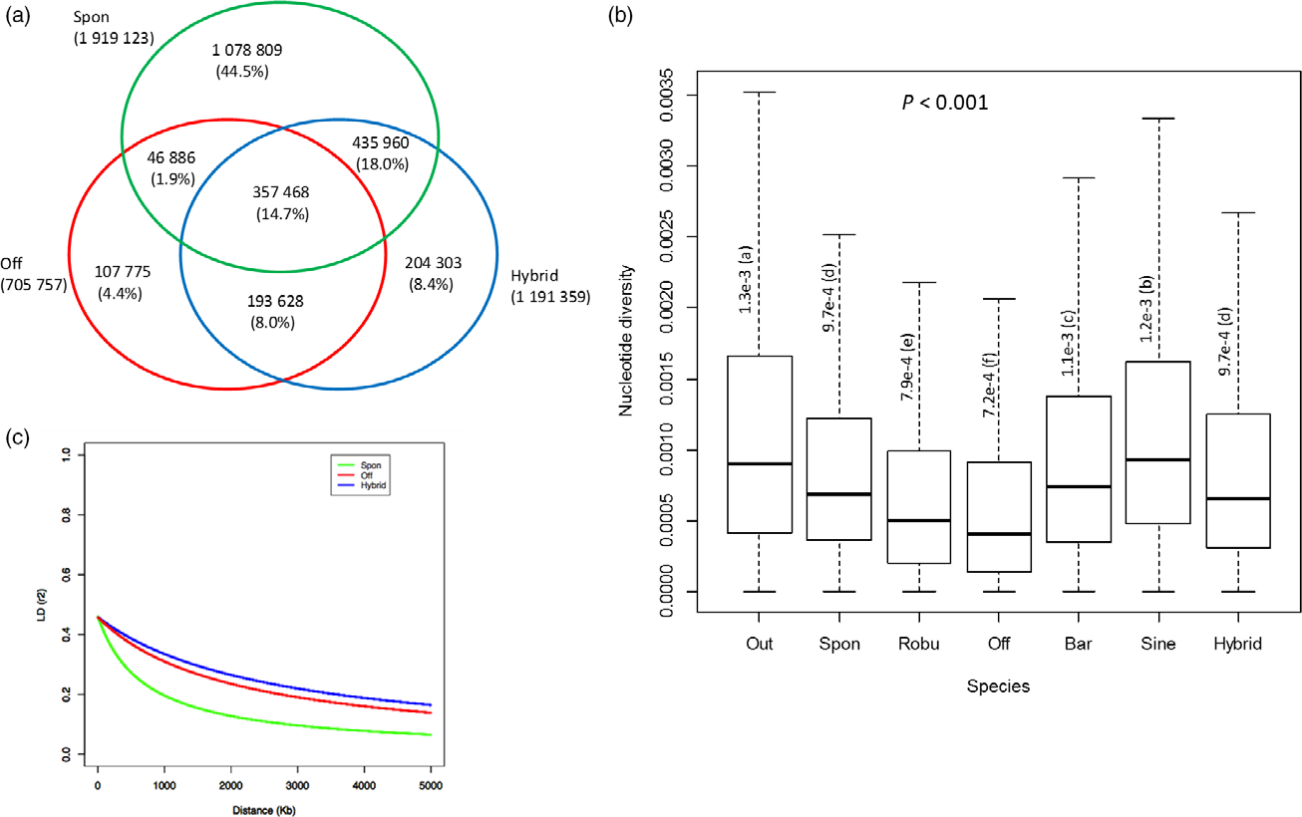
Statistics of sequence variations in the sugarcane diversity panel. (a) Venn diagram of SNPs identified in *S. spontaneum*,* S. officinarum* and modern *S*. hybrids. (b) Nucleotide diversity in *S. spontaneum*,* S. robustum*,* S. officinarum*,* S. barberi*,* S. sinence* and outgroup 2. (c) Decay of linkage disequilibrium (LD) in *S. spontaneum* (green), *S*. *officinarum* (red) and modern *S*. hybrids (blue). The decay of LD was measured by correlation coefficients (*r* ^2^). Spon = *S. spontaneum*; Off = *S*. *officinarum*; Hybrid = modern *S*. hybrids; Out = outgroup 2; Robu = *S. robustum*; Bar = *S. barberi*, Sine = *S. sinence*.

In the *Saccharum* spp SNP database, a total of 2.42 million SNPs were present in the three sub‐populations, of which 1.92, 0.71 and 1.19 million SNPs for *S*. *spontaneum*,* S*. *officinarum* and modern *S*. hybrids respectively (Figure 3a). The largest number of unique SNPs was for *S*. *spontaneum* (1.07 million), followed by modern *S*. hybrids (0.20 million) and *S*. *officinarum* (0.11 million). The number of SNPs shared between *S*. *spontaneum* and *S*. *officinarum*,* S*. *spontaneum* and modern *S*. hybrids, and *S*. *officinarum* and modern *S*. hybrids was 0.40 million, 0.79 million and 0.55 million respectively. Majority of the SNPs in *S. spontaneum* (58.7%) were not found in the modern *S*. hybrids. The percentages of SD SNPs were 62.6%, 58.4% and 63.0% for *S*. *spontaneum*,* S*. *officinarum* and modern *S*. hybrids respectively (Figure 3b). In addition to SNPs, 525.3 K InDels, 590 PAVs and 9,201 SSRs were present in the three sub‐populations. A total of 3039 species‐specific sequence variations were characterized between *S. spontaneum* and *S. officinarum*, including 1995 SNPs, 351 InDels, 33 PAVs and 660 SSRs.

The pair‐wise genetic distance between sugarcane hybrids (ancient and modern) and their progenitor species (*S. spontaneum* and *S. officinarum*) revealed potential ancestry contributor (or their close relatives) *S. spontaneum* and *S. officinarum* accessions to ancient and modern hybrids. For the ancient hybrids, the same four *S. spontaneum* accessions (Sample ID 45, 145, 230 and 278) with an origin of Indonesia were identified as potential ancestor accessions to both *S. barberi* and *S. sinense*. However, these two ancient hybrids had different *S. officinarum* accessions as potential ancestors. A *S. officinarum* accession (Sample ID 70) originated from Papua New Guinea was identified as the potential ancestry contributor to *S. barberi,* and two other different *S. officinarum* accessions (Sample ID 67 and 221) also originated from Papua New Guinea were identified as potential ancestry contributors to *S. sinense*. These results indicated that most likely that the two ancient sugarcane hybrids were derived from different hybridization events with the same *S. spontaneum* but different *S. officinarum* ancestry entries. For modern sugarcane hybrids, however, nine *S. spontaneum* (Sample ID 30, 45, 132, 145, 197, 230, 231, 254 and 278) and seven *S*. *officinarum* (Sample ID 99, 117, 154, 161, 188, 217 and 290) accessions were identified as ancestry entries suggesting a relatively wide genetic basis in modern hybrids influenced by diverse breeding programmes recently. Interestingly, it was noticed that the four *S. spontaneum* accessions as the ancestry contributors to both *S. barberi* and *S. sinense* were also included in the nine ancestry entries to modern sugarcane hybrids, although all their *S. officinarum* ancestry contributors were different. Therefore, it was speculated that the four *S. spontaneum* accessions originated from Indonesia played fundamental role in contributing the genetic background of stress tolerance to both ancient and modern sugarcane hybrids.

We further explored the genome compositions of ancient and modern hybrids using species‐specific SNPs identified between *S*. *spontaneum* and *S*. *officinarum*. The proportion of *S*. *officinarum* genome present in *S. barberi*,* S. sinense* and modern *S. *hybrids was significantly different with an average of 55.3% (51.7%−58.3%), 54.5% (53.2%−55.4%) and 69.1% (55.8%−79.2%) presence in *S. barberi*,* S. sinense* and modern hybrids, respectively. The *S*. *spontaneum* genome on the other hand contributed an average of 38.7% (36.3%−41.5%), 36.8% (34.5%−37.4%) and 26.3% (17.1%−36.8%) respectively (Figure S3c, d). The ancient hybrids had much higher genome contributions from *S*. *spontaneum* than modern *S*. hybrids (Figure 3a, and S3), which was consistent with phylogenetic analyses above. In addition, the overall nucleotide diversities were significantly different among *S. barberi*,* S. sinense* and modern *S. *hybrids (*P *<* *0.001) with overall nucleotide diversity of 1.1e^−3^, 1.2e^−3^ and 9.7e^−4^ respectively (Figure 3b). The drastic reduction in nucleotide diversity in modern hybrids demonstrated the influence of backcrossing process and a strong selection in sugarcane breeding programmes. The results further illustrated that the three hybrid groups differentiated due to different hybridization processes.

### Patterns of variation in *S*. *spontaneum, S*. *officinarum* and modern *S*. hybrids

We estimated LD decay with distance for the three sub‐populations, *S*. *spontaneum, S*. *officinarum* and modern *S*. hybrids, using high‐quality genome‐wide SNPs. A total of 55 184, 44 546 and 63 126 SNPs with one SNP per 13.3 Kbp, 16.4 Kbp and 11.6 Kbp monoploid genome of *S*. *spontaneum*,* S*. *officinarum* and modern *S*. hybrids, respectively, passed the filtering. On average, LD drops to 0.2 at 962.4 Kbp, 2739.2 Kbp and 3573.6 Kbp for *S*. *spontaneum*,* S*. *officinarum* and modern *S*. hybrids respectively (Figure 3c). The LD extent showed an increasing order in wild species, cultivated species and sugarcane hybrids. For each chromosome, the LD decay ranged from 803.9 to 1491.3 Kbp for *S*. *spontaneum*, 2025.9 to 3009.3 Kbp for *S*. *officinarum* and 3163.5 to 4451.0 Kbp for modern *S*. hybrids (Figure S4). Sugarcane exhibited a high LD extent, larger than that of most of the species analysed to date, such as 10 Kbp in *Arabidopsis thaliana* (Kim *et al*., 2007), ~150 Kbp in sorghum (Morris *et al*., 2013) and 420 Kbp in soybean (Zhou *et al*., 2015).

Pair‐wise genome‐wide fixation index (*F*st) values were calculated in the three sub‐populations. The results indicated a closer relationship between *S*. *officinarum* and modern *S*. hybrids (*F*st = 0.016) than between *S*. *spontaneum* and modern *S*. hybrids (*F*st = 0.040), and between *S*. *spontaneum* and *S*. *officinarum* (0.084), reflecting the impact of recurrent backcrosses in breeding programmes that enriched genomic background of *S*. *officinarum* in modern *S*. hybrids. The average nucleotide diversity was 9.7e^−4^, 7.2e^−4^ and 9.7e^−4^ for *S*. *spontaneum*,* S*. *officinarum* and modern *S*. hybrids respectively (Figure 3b). The nucleotide diversity for modern *S*. hybrids was at the same level with *S*. *spontaneum*, and both of them were significantly higher than that of *S*. *officinarum* (*P *<* *0.001). The genetic diversity of modern *S*. hybrids is most likely contributed by *S*. *spontaneum* even though modern *S*. hybrids inherited ~70% genome from *S*. *officinarum*. The significantly low nucleotide diversity of *S*. *officinarum* compared with *S*. *spontaneum* and its potential ancestor *S. robustum* (7.9e^−4^) reflects the potential of a genetic bottleneck during its domestication.

### Signature regions during sugarcane domestication and selection

**Figure 4:**
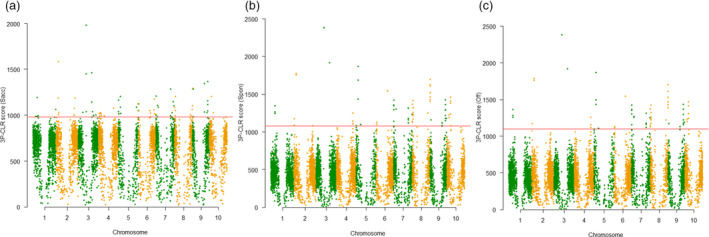
3P‐CLR scan of ancestor of *Saccharum* (a), *S. spontaneum* (b) and *S. officinarum* (c) in comparison with outgroup 2 revealed regions under selection. Spon = *S. spontaneum*; Off = *S*. *officinarum*; Sacc = ancestor of *Saccharum*. Red line showed the top 1% of scores in each scan.

To examine selective sweep in sugarcane genome, we performed a genome‐wide scan and selected the top 1% scores of the scanned intervals as candidate regions. Outgroup 2 (Figure 1b and Table S2) was used as the outgroup population for this analysis, which diverged from *Saccharum* ancestors after *Miscanthus* and was the closest group to *Saccharum* in this collection. In total, 58, 25 and 24 regions for the ancestor of *Saccahrum*,* S*. *spontaneum* and *S*. *officinarum* were identified as selective sweeps, respectively, with genome size ranging from 27.9 to 542.6 Kbp (Figure 4 and Table S5). After removing the completely redundant regions (most of the redundant regions were between *S*. *spontaneum* and *S*. *officinarum* branches), a total of 75 unique selective sweep regions were identified, which contained a total of 1815 genes under natural selection. By searching enriched Gene Ontology (GO) categories of the genes in the 75 selection sweep regions (Al‐Shahrour *et al*., 2004), two significant enriched categories, response‐to‐wounding and serine‐type endopeptidase inhibitor activity, were identified in the ancestor of *Saccahrum* branch (*P *<* *0.01 adjusted for FDR), which may contribute to the plant endurance during vegetative harvesting. Cell wall biogenesis and galactoside 2‐alpha‐L‐fucosyltransferase activity were enriched in both *S*. *spontaneum* and *S*. *officinarum* branches (*P *<* *0.001 adjusted for FDR), which may contribute to the increasing biomass of the two species.

**Figure 5:**
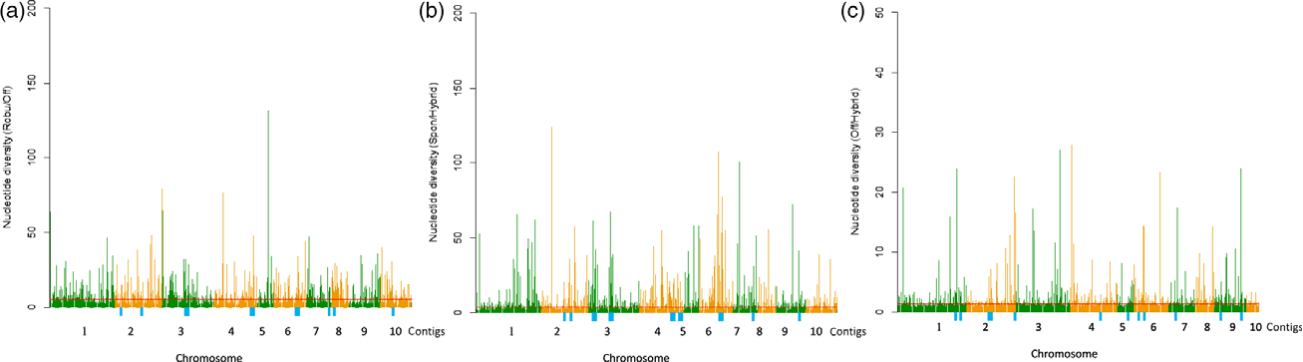
Ratio of nucleotide diversity for each target gene according to the sorghum genome. Ratio of nucleotide diversity between *S. robustum* and *S. officinarum* (a); *S*. *spontaneum* and modern *S*. hybrids (b); *S*. *officinarum* and modern *S*. hybrids (c). Each vertical line is a target gene, the solid red line shows the cut‐off of the top 5% ratio for each comparison, and the blue bars on the bottom indicated the candidate genes overlapped with sorghum orthologs that were homologous genes characterized as domestication or selection genes previously.

The nucleotide diversity of each gene between wild and cultivated accessions (or domesticated) can be compared to identify genes selected during domestication (or selection). Relatively low nucleotide diversity of the gene indicates its retention during domestication/selection. First, the nucleotide diversities of genes between *S*. *officinarum* and *S. robustum* were compared to identify the domestication genes as it was speculated that *S*. *officinarum* was domesticated from *S. robustum* (Brandes, 1958) and phylogenetic analysis in this study further validated this (Figure 1b). Of 12 981 genes surveyed, 649 genes showed relatively low nucleotide diversity and were considered as candidate domestication genes in *S*. *officinarum* (Figure 5a and Table S6). We further investigated these 649 domestication genes to see whether any of them were also identified in selective sweep intervals, and found 29, 11 and 10 genes were actually in the selective sweep interval from the ancestor of *Saccharum*,* S*. *spontaneum* and *S*. *officinarum* branch respectively (Figure S5a).

We further compared nucleotide diversities of each gene between *S. spontaneum* and modern *S*. hybrids, and between *S*. *officinarum* and modern *S*. hybrids to identify genes under selection in modern *S*. hybrids. Of 14 087 genes surveyed, 703 and 666 genes were identified as candidate genes selected from *S. spontaneum* and *S*. *officinarum*, respectively, into modern *S*. hybrids with 179 shared genes and 1190 non‐redundant selected genes (Figure 5, Tables S7, and S8). Interestingly, a total of 116 genes were common genes between the 649 domestication genes identified above and the 1190 non‐redundant selected genes (Figure S5b). We reviewed the 1190 non‐redundant selected genes and found 45, 13 and 12 genes were in the selective sweep intervals from ancestor of *Saccharum*,* S*. *spontaneum* and *S*. *officinarum* branch respectively (Figure S5c). All these common genes between domestication and selection, identified from different approaches, might have important roles in determining their species feature and contributing to robust agronomic traits for sugarcane cultivars. Therefore, a total of 1723 genes were identified as non‐redundant candidate genes, including 533 genes under domestication, 1074 genes under breeders’ selection in breeding programmes and 116 shared genes between domestication and selection.

We searched and curated 64 domestication and selection genes identified in other crop species, which corresponded to 50 sorghum orthologs (Table S9). We compared candidate regions or genes identified in our study with the curated genes by mapping them to the bins of the sorghum orthologs (50 Kbp upstream and downstream extension according to the sorghum genome). A total of 11 domestication genes and 29 selection genes were found in 10 and 19 curated sorghum ortholog regions, respectively (Figure 5), and eight of our selective sweep regions in the ancestor *Saccahrum* branch (Table S5) were in nine sorghum ortholog regions. These sorghum ortholog intervals harbouring the candidate genes in domestication, selection and sweeps had genes involved in flowering time, yield related traits and sugar metabolism. We closely examined one of the candidate genes (*Sobic.002G275100*), whose homolog in maize controlled flowering time at temperate latitudes (Lai *et al*., 2018). This gene showed strong species selection between non‐*Saccharum* and *Saccahrum* with 10 of 17 SNPs fixed at opposite alleles for the two species (Figure S6). The N/S ratio for this gene was 0.747, 0.467 and 0.500 for non‐*Saccharum*,* S*. *spontaneum* and *S*. *officinarum*, respectively, indicating its retention and stability in *Saccharum* spp. Significant reduction in nucleotide diversity was also observed in this gene between *S*. *spontaneum* and modern *S*. hybrids (6.8e^−4^ vs. 1.5e^−4^). The quick selection of specific alleles for this gene might have positively contributed to sugarcane synchronic hybridization and seed production in modern *S*. hybrids derived from sugarcane breeding programmes worldwide.

### Environmental association analysis

Geographic origins of 210 accessions in the diversity panel were retrieved for environmental association analysis (EAA). A total of 74 modern *S*. hybrids were not included for EAA as they were developed recently and possibly were not adapted to local environments completely yet. Based on the geographic origins, 104 environmental variables were obtained from the WORLDCLIM database (Table S10) (Fick and Hijmans, 2017) for the 136 accessions, and most of them were highly correlated (Figure S7a). We used principal components analysis (PCA) to extract top five principle components (PCs) for EAA, which could explain 89.2% variations for all of the environmental variables (Figure S7b). In total, 105 653 common bi‐allelic SNPs (MAF ≥ 0.05, call rate ≥95%) with 94 464 SNPs (89.4%) located in 12,246 genes were used for EAA. As a result, a total of 93, 17, 9 and 3 SNPs were significantly associated with PC1, PC2, PC3 and PC5 respectively (*P *=* *0.01 after Bonferroni correction) (Figure S7c, d, e, f and Table S11) with one common SNP (chr10:55153692) identified for both PC1 and PC2, thus a total of 121 SNPs were associated with four PCs, among which, 112 were located in 106 gene models. Functional enrichment analysis revealed that eight genes with function of proteolysis were significantly enriched in the 106 genes (*P *=* *4.27e^−4^). By manually checking the orthologs of the eight genes in Phytozome (http://www.phytozome.net/), the orthologs of two genes (*Sobic.003G199200* and *Sobic.010G208400*) co‐expressed with genes under high light conditions, which corroborated that the hydrolysis of proteins into smaller polypeptides and/or amino acids may play an important role in local adaptation, in agreement with previous reports that amino acids play important signalling roles for environmental adaptation (Rai, 2002).

The 106 genes related to the environmental adaption were further compared with candidate genes identified to be related to domestication and selection. We found 11 and 4 of the 106 genes were also identified by 3P‐CLR analysis and nucleotide diversity comparison respectively. Interestingly, the gene (*Sobic.005G034300*) annotated to function in assisting assembly of single‐chain polypeptides or multi‐subunit complexes into the correct tertiary structure (Lyne *et al*., 2015) is not only a gene associated with environmental adaptation but also related with domestication. We further noticed that this gene (*Sobic.005G034300*) co‐expressed with 163 genes (Lyne *et al*., 2015), which were enriched in several pathways including ribosome biogenesis, RNA degradation, protein processing, protein export and proteasome (*P *<* *0.001) (Kanehisa and Goto, 2000; Yi *et al*., 2013), suggesting a big network of genes involved in dynamic and active protein synthesis and processing contributed to adaptation of local environments and were fundamental for sugarcane domestication as well.

## Discussion

Genomic resources are critical tools and reservoir for molecular breeding and crop improvement. Sugarcane is a polyploid species (up to 12×) with high heterozygosity, which requires intensive sequencing depth to call genotypes accurately, thus has been a crop with “bitter” genome to study with. To separate SD SNPs from homozygote for dodeca‐ploid species, up to 108× sequence depth is required (Yang *et al*., 2017). In this study, we deeply sequenced targeted regions of a sugarcane diversity panel including 307 accessions, representing wide genetic diversity and geographical distribution of sugarcane germplasm in the world collection. A total of 4.65 million SNPs were identified, which compiled a novel and comprehensive sequence variation database for *Saccharum* spp. Moreover, with an average of 98× sequence depth, we are able to characterize 4.26 million SNPs with high‐confidence dosage resolution, which would be difficult to achieve by using other NGS approach. This SNP dataset with accurate genotype would be the basis for population genomics analyses in sugarcane, such as genome‐wide association studies (GWAS), gene mapping and so on. We also characterized 3,039 species‐specific sequence variations between *S. spontaneum* and *S. officinarum*, which can serve as diagnostic tools to fingerprint germplasm and estimate genome composition of clones. Based on the genome‐wide SNPs, we comprehensively evaluated genetic relationships of the sugarcane diversity panel and noticed a few discrepancies of naming between the WCSRG records and our results. The WCSRG records were based on traditional taxonomic methods using morphological characteristics, which can be seriously impacted by plant growth conditions. Particularly, the *S*. *officinarum* and modern *S*. hybrids had highly similar genome background, and the traditional taxonomic methods were often difficult to discriminate them. All seven non‐*Saccharum* species in outgroup 2 were found to be synonymous with *Erianthus* species (The Plant List, 2010; USDA and NRCS, 2016). Accessions of this group were most likely *Erianthus* New World Species, which were genetically similar to sugarcane (Jackson and Henry, 2011). In this study, we placed several unknown accessions and assigned misclassified accessions into specific groups that they should belong to.

**Figure 6:**
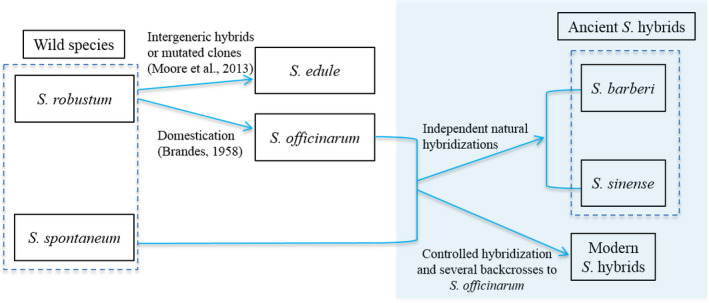
Evolutionary relationships of *Saccharum* genus. The rectangle shaded with light blue showed relationships supported by this study.

We proposed an evolutionary relationship for *Saccharum* genus (Figure 6) based on literature and our findings. We believe that the two ancient and modern sugarcane hybrids have very different origins with different ancestry contributing accessions, specifically *S. officinurum*. We observed a bottleneck of genetic diversity in *S. officinarum*, which reflected a very strong selection in *S. officinarum* during its domestication. This explained evolutionary driving force of frequent natural hybridizations between *S. officinarum* and other closely related species, such as *S. spontaneum*. Multiple evidences support that ancient and modern hybrids derived from the hybridization between *S. spontaneum* and *S. officinarum* (D'Hont *et al*., 2002; Irvine, 1999; Nair *et al*., 1999), although there were some debates regarding whether the two ancient hybrids come from single origination. Our results clearly showed that *S. sinense* and *S. barberi* were different in terms of genome compositions and potential ancestor accessions, specifically they had different *S. officinarum* accessions as potential ancestor in the hybridization. The natural hybridization probably happened in the same sites while *S. officinarum* accessions were brought in. We also reconstructed pedigrees for modern *S*. hybrids in the sugarcane diversity panel. The results emphasized again that a few foundation accessions involved in the hybridizations in the breeding programme as previous reports (Acevedo *et al*., 2017; Deren, 1995; Lima *et al*., 2002). The narrow genetic basis in modern sugarcane hybrids may raise the risk of outbreak of new diseases. This study highlighted the urgency and importance of broadening the genetic resources in sugarcane breeding programmes.

For the first time, we evaluated LD decay in *S. spontaneum*,* S. officinarum* and *S*. hybrids using a model specifically designed for polyploid species and genome‐wide SNPs included dosages into consideration. We observed extremely high LD extents in the three sub‐populations of sugarcane compared with many other plant species with estimated LD (Kim *et al*., 2007; Morris *et al*., 2013; Zhou *et al*., 2015). The high LD extent in sugarcane is mostly caused by the long‐life cycle (~12 months), perennial nature and readily clonal propagation feature of sugarcane clones, which would significantly reduce the frequency of chromosome recombination events. As a result, clustered genes controlling multiple traits in sugarcane could be selected during domestication and hybridization. Therefore, in the genome scanning, large selective regions were observed in sugarcane with an average of 280.4 Kbp in size, which contained significant number of genes (1815) under natural selection. With this high LD extent, high marker density may not be very critical for performing GWAS or related studies in sugarcane. However, breaking linkage drag and performing gene fine mapping, or map‐based cloning, would be challenging in sugarcane through forward genetics approach in addition to the challenges added by high level of polyploid.

Scanning the genome is the most efficient approach to quickly identify loci under adaptation or selection, especially with phenotypes hard to be thoroughly scored (Flood and Hancock, 2017). In this study, the genome‐wide scanning for selective sweeps and nucleotide diversity comparison in *Saccharum* sub‐populations allowed us to identify 75 selective sweeps under natural selection and 1723 candidate genes involved in domestication and breeding selection. A large number of genes under domestication or selection were also identified in maize (Hufford *et al*., 2012), suggesting that thousands of genes with diverse function must be needed to shape agronomically productive crops with long breeding and selection history due to its relative large LD. A total of 8 selective sweeps, 11 domestication genes and 29 selection genes in breeding programmes were overlapped with curated genes characterized in other crops, supporting the power of this analysis to identify signature sequence variations during historical evolutions. Moreover, novel domestication and selection genes identified in this study could serve as a candidate gene reservoir for further gene characterization and validation in sugarcane and related crop species. In addition, we identified 121 SNPs landed in 106 gene regions significantly associated with environmental variables. Gene enrichment analysis of associated genes suggested that genes encoding protein hydrolysis might be important for local environmental adaptation.

## Experimental procedures

### Plant materials and DNA extraction

A diversity panel consisting of 299 accessions (Nayak *et al*., 2014) selected from the WCSRG maintained at the USDA‐ARS Subtropical Horticulture Research Station, Miami, Florida and eight modern sugarcane hybrids frequently used as parental materials in the USA Florida sugarcane breeding programme (Table S1 and Figure S1) were included in this study. In total, 307 accessions were established at USDA‐ARS Sugarcane Field Station at Canal Point, FL in 2013 in a field nursery. Young leaves were collected from each accession for DNA extraction using the cetyltrimethyl ammonium bromide (CTAB) method (Wang *et al*., 2010).

### Sequence analyses

The target enrichment sequencing of each accession was conducted according to Song *et al*. (2016). Clean reads longer than 50 bp were aligned to sorghum genome v3.0 (Paterson *et al*., 2009) using BWA‐mem (Li, 2013) with default settings. Uniquely mapped reads were extracted, and sequence variants (SNP and InDels) were called using Unified Genotyper implemented in Genome Analysis Tool Kit v3.30 (McKenna *et al*., 2010). Details of library preparation and sequencing, sequence variation calling, and PAV and SSR identification were presented (Data S2).

We used phylogenetic analysis to infer genetic relationships among the diversity panel using Molecular Evolutionary Genetics Analysis version 6.0 (Tamura *et al*., 2013). Genetic structure was further assessed with default settings by DAPC implemented in the adegent package for R (Jombart, 2008; Jombart *et al*., 2010) and ADMIXTURE v1.30 (Alexander *et al*., 2009). Linkage disequilibrium (correlation coefficient values (*r* ^2^)) was calculated using SHEsisPlus (Shen *et al*., 2016) for *S*. *spontaneum*,* S*. *officinarum* and modern *S. *hybrids respectively. Pair‐wise *F*st among the three sub‐populations were calculated using the method described (Nei, 1973). Nucleotide diversity (π) per site was calculated for each sub‐population (Begun *et al*., 2007). A genome scan of selective sweeps was performed using 3P‐CLR (Racimo, 2016). We focused on the sub‐populations *S. spontaneum* and *S. officinarum* using non‐*Saccharum* as the outgroup population. Likelihood‐ratio statistics were calculated by a sliding‐window approach with a central SNP in every 20 SNPs. For each scan, we selected the windows in the top 1% of scores as candidate selective sweeps. More details can be found online Data S2.

### Environmental association analysis

Association between SNPs and bioclimatic variables was analysed in 136 unrelated individuals in the diversity panel with sampling location information. Climate data for current conditions (1970–2000) were obtained from the WORLDCLIM database (Fick and Hijmans, 2017) at a resolution of 10 min (~340 km^2^). The EAA was performed with LFMM program (Frichot *et al*., 2013) for the top five principal components (PCs). In brief, the EAA was performed 10 independent times for each PC for a total of 10 000 MCMC cycles, with 5000 burn‐in cycles. Then z‐scores from the 10 runs were combined and a *P*‐value for the association between markers and PCs was calculated following the LFMM manual (Frichot *et al*., 2013). Details of environmental association analysis were presented in online Data S2.

### Data access

The cleaned target‐enrichment sequencing reads generated in this study were deposited into NCBI Short Reads Archive with an accession number of SRP132365. A catalogue of *Saccharum* spp sequence variation database was deposited into Gatorcloud (https://bit.ly/2zKQXhE). The rest intermediate analysis data and the plant materials will be available upon request.

## Supporting information


**Figure S1** An overview of the sugarcane diversity panel.
**Figure S2** Ancestry coefficient bar plots for an assumed number of sub‐populations (K) from three to eight.
**Figure S3** Statistics of species‐specific SNPs in ancient hybrids, *S. barberi*,* S. sinense* and modern *S. *hybrids.
**Figure S4** Linkage disequilibrium (LD) determined by squared correlation coefficient (*r* ^2^) against distance for each chromosome according to the sorghum genome (a), (b), (c), (d), (e), (f), (g), (h), (i) and (j) in *S. spontaneum* (green), *S*. *officinarum* (red) and modern *S*. hybrid (blue).
**Figure S5** Number of domestication and selection genes identified in this study Venn diagram of domestication genes and candidate genes in selective sweep intervals identified for the ancestor of *Saccharum*,* S*. *spontaneum* and *S*. *officinarum* branch respectively (a); domestication genes and selection genes identified from *S. spontaneum* and modern *S*. hybrids, and *S*. *officinarum* and modern *S*. hybrids comparisons (b); Selection genes and candidate genes in selective sweep intervals identified for the ancestor of *Saccharum*,* S*. *spontaneum* and *S*. *officinarum* branch respectively (c).
**Figure S6** Allele frequencies of alternative allele at each SNP locus in gene *Sobic.002G275100*.
**Figure S7** Summary of environmental association analyses (EAA).

**Table S1** Details on 299 sugarcane accessions plus 8 modern *S*. hybrids.
**Table S2** Statistical summary of target‐enrichment sequencing data in the diversity panel.
**Table S3** Statistical summary of single‐nucleotide polymorphisms (SNPs) according to sorghum genome.
**Table S4** Representative accessions selected based on phylogenetic analysis and structure analyses.
**Table S5** Selective sweep detect by 3P‐CLR in ancestor of *Saccharum* (Sacc), *S. spontaneum* (Spon) and *S*. *officinarum* (Off) branch.
**Table S6** Nucleotide diversity (π) of domestication genes identified by comparison of *S. robustum* (Robu) and *S. officinarum* (Off).
**Table S7** Nucleotide diversity (π) of selection genes identified by comparison of *S. spontaneum* (Spon) and modern *S*. hybrids (Hybrid).
**Table S8** Nucleotide diversity (π) of candidate genes under selection identified by comparison of *S. officinarum* (Off) and modern *S*. hybrids (Hybrid).
**Table S9** Candidate genes in domestication or selection from literature.
**Table S10** A total of 104 environmental variables in the diversity panel.
**Table S11** SNPs and genes associated with the five principal components (PCs).

**Data S1** Fasta Sequences of 60 000 probes used in this study.
**Data S2** Supporting experimental procedures.
