# Supplementary material for: Target enrichment sequencing of 307 germplasm accessions identified ancestry of ancient and modern hybrids and signatures of adaptation and selection in sugarcane (Saccharum spp.), a ‘sweet’ crop with ‘bitter’ genomes
Source: Plant Biotechnol J. 2018 Aug 29;17(2):488–98. doi: 10.1111/pbi.12992 (PMC6335080; doi:10.1111/pbi.12992)

**Supporting experimental procedures**

**DNA extraction and sequencing**

Young leaves were collected from each accession, and the DNA was extracted by using the cetyltrimethyl ammonium bromide (CTAB) method (Wang et al., 2010). The target enrichment sequencing of each accession was conducted (Song et al., 2016). In brief, 120-bp oligonucleotides that served as probes were designed to capture specific regions including candidate genes and well–distributed coding regions according to sorghum genome v3.0 (Paterson et al., 2009). A total of 50,935 probes were adopted from Song’s report (Song et al., 2016) and 9,065 were newly supplemented. In total, a set of 60k RNA probes (Supplemental file 1) were used for target enrichment sequencing in this study. DNA concentration was detected using Quant-iT PicoGreen dsDNA assay kit (Life Technologies, Carlsbad, CA). A total of 1 μg of genomic DNA of each accession was fragmented into 400bp fragments by using Covaris E220 Ultrasonicator (Covaris, Woburn, MA), and purified with Agencourt Ampure beads (Beckman Coulter Life Sciences, Indianapolis, IN). The adapters harboring barcodes were ligated to sheared DNA fragment after ends repairing, A-tail addition, and amplification for gDNA library preparation was done according to the product manual of Agilent SureSelect^XT^ reagents (Agilent Technologies, Santa Clara, CA). The quality and quantity of the prepped DNA libraries were assessed with a Bioanalyzer 2100. A set of 24 libraries each were pooled in equimolar concentration for target enrichment by using SureSelect Target Enrichment kit (Agilent Technologies, USA) with a total of 60k 120-bp length RNA probes. The captured DNA libraries were indexed and cleaned using Ampure beads and quantified by qPCR using KAPA Library Quantification Kits (KAPA Biosystems.com) and sequenced in paired-end mode by Illumina HiSeq 2000.

**Reads alignment and sequence variation calling**

The raw reads were sorted into reads per library according to their barcode sequences and then the barcodes were removed from raw data. The paired-end reads were trimmed from ends in pairs based on PHRED score of 20 using Trimommatic v0.36 (Bolger et al., 2014). Reads longer than 50 bp were aligned to sorghum genome v3.0 (Paterson et al., 2009). The reads containing both ends of the sequences were synchronized to keep the same order for paired-end alignment mode. The unpaired singleton reads that lost their mates after quality trimming were aligned as single-end reads. BWA-mem (Li, 2013) with default settings was used for alignment. Uniquely mapped reads were extracted from sequence alignment BAM files using mapping quality >0. The BAM files from both single-end mode and paired-end mode were merged to perform variant calling. The coverage of target regions was investigated by BEDTools (Quinlan and Hall, 2010).

SNPs were called using Unified Genotyper implemented in Genome Analysis Tool Kit (GATK) v3.30 (McKenna et al., 2010) with settings: -ploidy (ploidy) -mbq 20 -mgl 20 -stand_call_conf 30 -stand_emit_conf 10. The original SNPs were subjected to two levels of filtering for each individual accession. Criteria for level 1 filtering were: 1) mapping quality for SNPs > 30; 2) base quality for SNP sites > 20; 3) for a heterozygous genotype, at least two reads for minor allele were required; otherwise, missing genotypes were assigned; 4) for a given SNP locus with homozygous genotype, minimum reads were calculated according to ploidy of accessions following Yang et al. to ensure single dose SNPs were not called as homozygotes (Yang et al., 2017). Based on the calculation, minimum reads of 5, 11, 17, 23, 29 and 35 were required for ploidy at 2, 4, 6, 8, 10 and 12 for homozygous genotype calling, respectively; otherwise, missing genotypes were assigned. SNPs after level 1 filtering were documented as *Saccharum* spp. SNP database. After level 1 filtering, SNPs were further subjected to level 2 filtering to ensure accuracy of SNP genotype (or dosage) calling. According to the assumption that the number of reads for alleles at a SNP locus follow a binomial distribution (Li et al., 2014), the probability of SNP genotype can be calculated for all dosages at any sequencing depth. We calculated sequencing depth required to separate all SNP genotypes with a probability higher than 95% at determined ploidy, which were 31, 47, 70, 90 and 108 for ploidy at 4, 6, 8, 10 and 12, respectively. The SNPs after level 2 filtering were documented as SNP genotypes of the diversity panel. InDels were called using GATK and filtered following SNP level 1 described above. Species-specific SNPs and InDels between *S*. *spontaneum* and *S*. *officinarum* were characterized using bi-allelic SNPs and InDels with a call rate (> 80%) and fixed at alternative allele (allele frequency (AF) > 90%) (fixed as reference allele for the other species).

Presence/absence variants (PAVs) in the diversity panel were identified according to the uniquely mapped reads, which were aligned to probe designed regions with an extension of 200 bp upstream and downstream of the target regions. The PAVs were characterized following the criteria: 1) absence was called when no reads aligned to target regions; 2) presence was called if at least 10 clean reads aligned to target regions; and 3) missing genotypes were assigned for accessions with less than 10 aligned reads. Only accessions with at least three million uniquely mapped reads were included in this analysis to eliminate absence called due to low sequencing depth. Species-specific PAVs were defined as: 1) *S. spontaneum* specific PAVs were characterized as absence in all accessions of *S*. *spontaneum* and presence in at least 10 accessions of *S*. *officinarum*; 2) *S. officinarum* specific PAVs were characterized as absence in all accessions of *S*. *officinarum* and presence in at least 10 accessions of *S*. *spontaneum*.

To identify simple sequence repeats (SSRs), we assembled the clean reads into contigs for each accession using SPAdes3.10.0 (Bankevich et al., 2012) with default settings. The assembled contigs for each accession were used to mine SSRs using MIcroSAtellite identification tool (MISA) (<http://pgrc.ipk-gatersleben.de/misa/>). Contigs with putative SSRs were mapped to the sorghum genome. Only SSRs that can be uniquely mapped to the sorghum genome were further analyzed. Species-specific SSRs between *S*. *spontaneum* and *S*. *officinarum* were characterized as follows: 1) SSRs were detected in at least 10 accessions of one species but not in all accessions of the other species; or 2) SSRs were detected in at least 10 accessions of both groups but with different lengths.

**Population genomic analyses**

We used phylogenetic analysis to infer genetic relationships among the diversity panel using Molecular Evolutionary Genetics Analysis version 6.0 (MEGA 6.0) (Tamura et al., 2013). The pair-wise genetic distance for the 307 accessions was obtained from MEGA 6.0. Genetic structure was further assessed with default settings by DAPC implemented in the adegent package for R (Jombart, 2008; Jombart et al., 2010), and ADMIXTURE v1.30 (Alexander et al., 2009). The best group number was chosen with a minimized Bayesian Information Criterion (BIC) and cross-validation error for DAPC and ADMIXTURE, respectively.

Linkage disequilibrium (LD) (correlation coefficient values (r^2^)) was calculated using SHEsisPlus (Shen et al., 2016) for *S*. *spontaneum*, *S*. *officinarum* and modern *S.* hybrids, respectively. SHEsisPlus is a toolset designed for genetic studies for polyploid species, in which LD can be analyzed under polyploidy model. For each species, only accessions with the same ploidy were included in the analysis. SNPs after level 2 filtering with less than 20% missing data and minor allele frequency (MAF) higher than 5% were used for this analysis. LD between SNP pairs within the same chromosome was calculated using a 5 Mbp window. LD decay with distance was estimated following the Hill and Weir formula (Hill and Weir, 1988).

Pair-wise *F*st among the three sub-populations were calculated using the method described (Nei, 1973). Nucleotide diversity (π) per site was calculated for each sub-population (Begun et al., 2007). Nucleotide diversity was analyzed following the criteria: 1) Only SNPs with call rates higher than 80% were used; 2) at least five SNPs were required to calculate nucleotide diversity for the target gene. SNPs after level 2 filtering and with call rate ≥ 80% were used for *F*st and nucleotide diversity analysis. For comparison between sub-populations, we used the top 5% of surveyed genes as the cutoff to identify genes with differential nucleotide diversity.

**Genome scanning for selective signals**

A genome scan of selective sweeps was performed using 3P-CLR (Racimo, 2016). We focused on the sub-populations *S. spontaneum* and *S. officinarum* using non *Saccharum* as the out-group population. SNPs after level 2 filtering with call rate ≥ 80% and not fixed in the out-group population were used for the 3P-CLR analysis. Genetic distance was converted according to physical distance by assuming uniform recombination. Likelihood-ratio statistics were calculated by a sliding-window approach with a central SNP in every 20 SNPs. The window size was set to 0.25 cM and 100 SNPs were randomly sampled from each window. For each scan, we selected the windows in the top 1% of scores as candidate selective sweeps.

**Environmental association analysis**

Association between SNPs and bioclimatic variables was analyzed in 136 unrelated individuals in the diversity panel with sampling location information. Climate data for current conditions (1970–2000) were obtained from the WORLDCLIM database (Fick et al., 2017) at a resolution of 10 minutes (~340 km^2^). These data included minimum, maximum and average temperature (°C), average precipitation (mm), solar radiation (kJ m^-2^ day^-1^), wind speed (m s^-1^), water vapor pressure (kPa) for each month, and 19 bioclimatic variables derived from monthly temperature and precipitation. Including altitude, a total of 104 climate variables was included into this analysis. Due to high correlation between environmental variables and plants are responsible to environmental stimuli as a whole, we conducted a principal components analysis (PCA) to condense these environmental variables to new synthetic environmental factors with Factoextra R Package (Kassambara and Mundt 2016). The EAA was performed with LFMM program (Frichot et al., 2013) for the top five principal components (PCs). In brief, the EAA was performed 10 independent times for each PC for a total of 10,000 MCMC cycles, with 5,000 burn‐in cycles. Then z‐scores from the 10 runs were combined and a p‐value for the association between markers and PCs were calculated following the LFMM manual (Frichot et al., 2013).

**References**

Alexander, D.H., Novembre, J., and Lange, K. (2009). Fast model-based estimation of ancestry in unrelated individuals*. Genome Res.* **19**, 1655-1664.

Bankevich, A., Nurk, S., Antipov, D., Gurevich, A.A., Dvorkin, M., Kulikov, A.S., Lesin, V.M., Nikolenko, S.I., Pham, S., and Prjibelski, A.D. (2012). SPAdes: a new genome assembly algorithm and its applications to single-cell sequencing*.* *J. Comput Biol*. **19**, 455-477.

Begun, D.J., Holloway, A.K., Stevens, K., Hillier, L.W., Poh, Y., Hahn, M.W., Nista, P.M., Jones, C.D., Kern, A.D., and Dewey, C.N. (2007). Population genomics: whole-genome analysis of polymorphism and divergence in Drosophila simulans*. PLoS boil*. **5**, e310.

Bolger, A.M., Lohse, M., and Usadel, B. (2014). Trimmomatic: a flexible trimmer for Illumina sequence data*. Bioinformatics* **30**, 2114-2120.

Fick, S.E., and Hijmans, R.J. (2017). WorldClim 2: new 1‐km spatial resolution climate surfaces for global land areas. *Int. J. Climatol.* **37**, 4302-4315.

Frichot, E., Schoville, S.D., Bouchard, G., and François, O. (2013). Testing for associations between loci and environmental gradients using latent factor mixed models. *Mol. Biol. Evol.* **30**, 1687-1699.

Hill, W.G., and Weir, B.S. (1988). Variances and covariances of squared linkage disequilibria in finite populations*. Theor. Popul. Biol.* **33**, 54-78.

Kassambara, A., and Mundt, F. (2016). Factoextra: extract and visualize the results of multivariate data analyses. R Package Version 1.

Li, H. (2013). Aligning sequence reads, clone sequences and assembly contigs with BWA-MEM*.* arXiv preprint arXiv:1303.3997.

Li, X., Wei, Y., Acharya, A., Jiang, Q., Kang, J., and Brummer, E.C. (2014). A saturated genetic linkage map of autotetraploid alfalfa (*Medicago sativa* L.) developed using genotyping-by-sequencing is highly syntenous with the *Medicago truncatula* genome*. G3* (Bethesda, Md.) **4**: 1971-1979.

Jombart, T. (2008). adegenet: a R package for the multivariate analysis of genetic markers*. Bioinformatics* **24**, 1403-1405.

Jombart, T., Devillard, S., and Balloux, F. (2010). Discriminant analysis of principal components: a new method for the analysis of genetically structured populations*. BMC Genet.* **11**, 94

McKenna, A., Hanna, M., Banks, E., Sivachenko, A., Cibulskis, K., Kernytsky, A., Garimella, K., Altshuler, D., Gabriel, S., and Daly, M. (2010). The Genome Analysis Toolkit: a MapReduce framework for analyzing next-generation DNA sequencing data*. Genome Res* **20**, 1297-1303.

Nei, M. (1973). Analysis of gene diversity in subdivided populations*.* *Proc. Natl Acad. Sci.* **70**, 3321-3323.

Paterson, A.H., Bowers, J.E., Bruggmann, R., Dubchak, I., Grimwood, J., Gundlach, H., Haberer, G., Hellsten, U., Mitros, T., Poliakov, A., et al. (2009). The Sorghum bicolor genome and the diversification of grasses*. Nature* **457**, 551-556.

Quinlan, A.R., and Hall, I.M. (2010). BEDTools: a flexible suite of utilities for comparing genomic features*.* *Bioinformatics* **26**: 841-842.

Racimo, F. (2016). Testing for ancient selection using cross-population allele frequency differentiation*. Genetics* **202**, 733-750.

Shen, J., Li, Z., Chen, J., Song, Z., Zhou, Z., and Shi, Y. (2016). SHEsisPlus, a toolset for genetic studies on polyploid species*. Sci. Rep*. **6**.

Song, J., Yang, X., Resende, M.F., Neves, L.G., Todd, J., Zhang, J., Comstock, J.C., and Wang, J. (2016). Natural allelic variations in highly polyploidy *Saccharum* complex*. Front. plant Sci.* **7**.

Tamura, K., Stecher, G., Peterson, D., Filipski, A., and Kumar, S. (2013). MEGA6: molecular evolutionary genetics analysis version 6.0*. Mol. Biol. Evol.* **30**, 2725-2729.

Wang, J., Roe, B., Macmil, S., Yu, Q., Murray, J.E., Tang, H., Chen, C., Najar, F., Wiley, G., Bowers, J., et al. (2010). Microcollinearity between autopolyploid sugarcane and diploid sorghum genomes*. BMC Genom*. **11**, 261.

Yang, X., Song, J., You, Q., Paudel, D.R., Zhang, J., and Wang, J. (2017). Mining sequence variations in representative polyploid sugarcane germplasm accessions*. BMC Genom*. **18**, 594.

**Figure S1.** An overview of the sugarcane diversity panel. (a) The representativeness of the 299 selected accession from the World Collections of Sugarcane and Related Grasses (WCSRG). The 299 selected accessions were labeled in blue color and the rest of unselected accessions were in gray color. The phylogenetic tree was conducted based on 209 SSR alleles in assessing the 1,002 accessions in WCSRG (Nayak *et al.*, 2014). (b) Pedigrees of modern *S*. hybrids based on literature search and breeding programs’ records. The size of circle reflects contributions of the clones in sugarcane breeding programs.


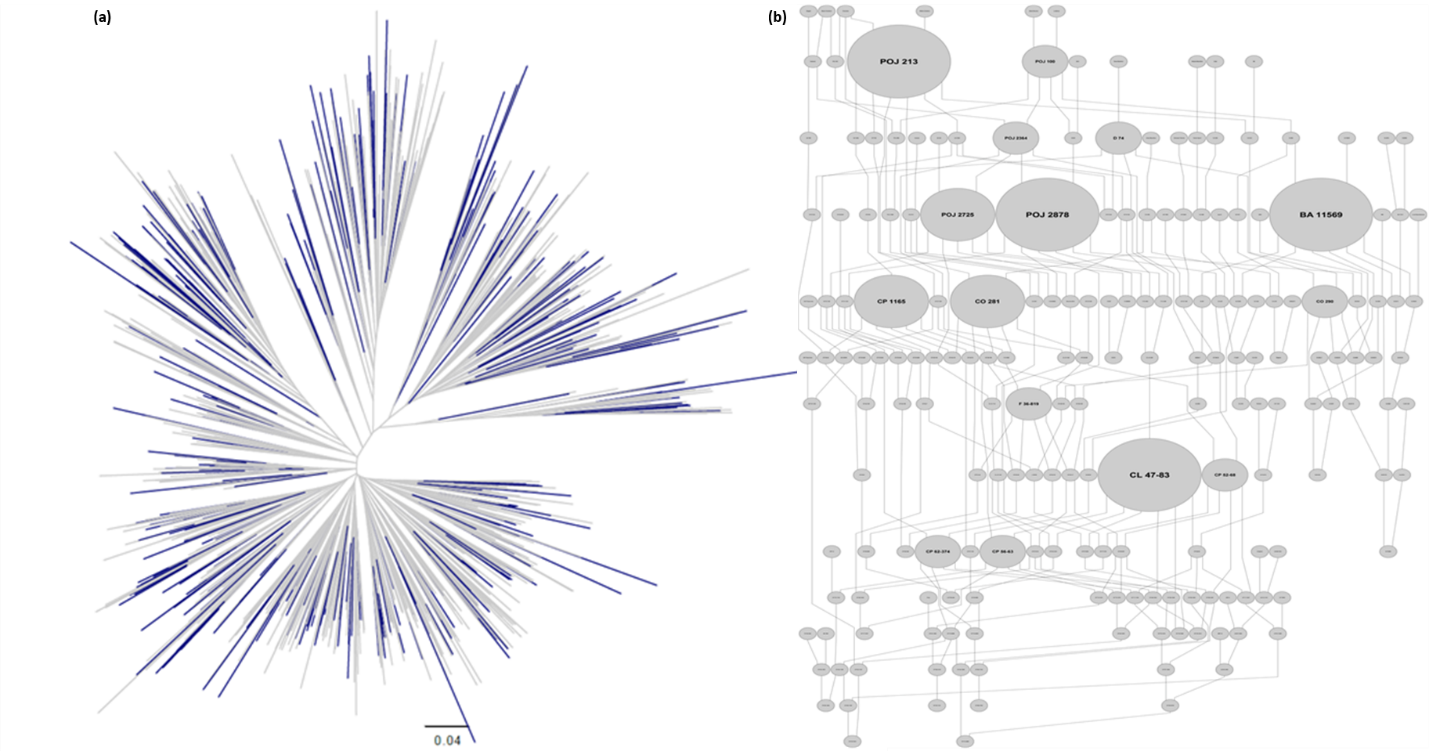


**Figure S2.** Ancestry coefficient bar plots for an assumed number of subpopulations (K) from three to eight. Robu = *S. robustum*; Spon = *S. spontaneum*; Off = *S. officinarum*; Hybrid = modern *S.* hybrids; Barb = *S. barberi*, Sine = *S. sinence*; Non sacc = Non *saccharum*.


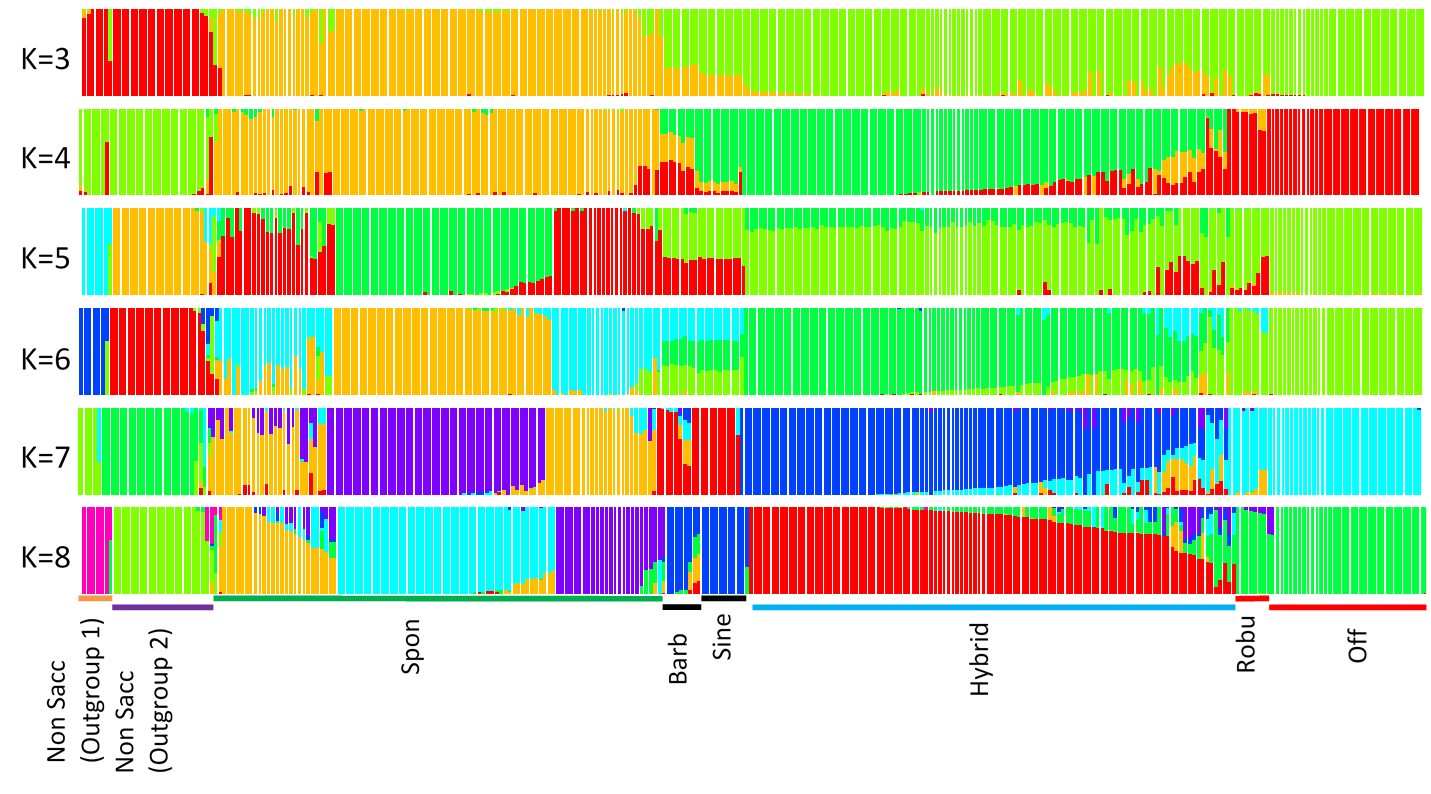


**Figure S3.** Statistics of species-specific SNPs in ancient hybrids, *S. barberi*, *S. sinense*, and modern *S.* hybrids. Venndiagram of SNPs identified in *S. spontaneum*, *S. officinarum*, and *S. barberi* (a); *S. spontaneum*, *S. officinarum*, and *S. sinense* (b); (c) Proportion of *S. officinarum* genome inherited in *S. barberi*, *S. sinense* and *S.* hybrids based on species-specific SNPs; (d) Proportion of *S. spontaneum* genome inherited in *S. barberi*, *S. sinense* and *S.* hybrids. Spon = *S. spontaneum*, Off = *S. officinarum*, Barberi = *S. barberi* and Sinense = *S. sinense*.


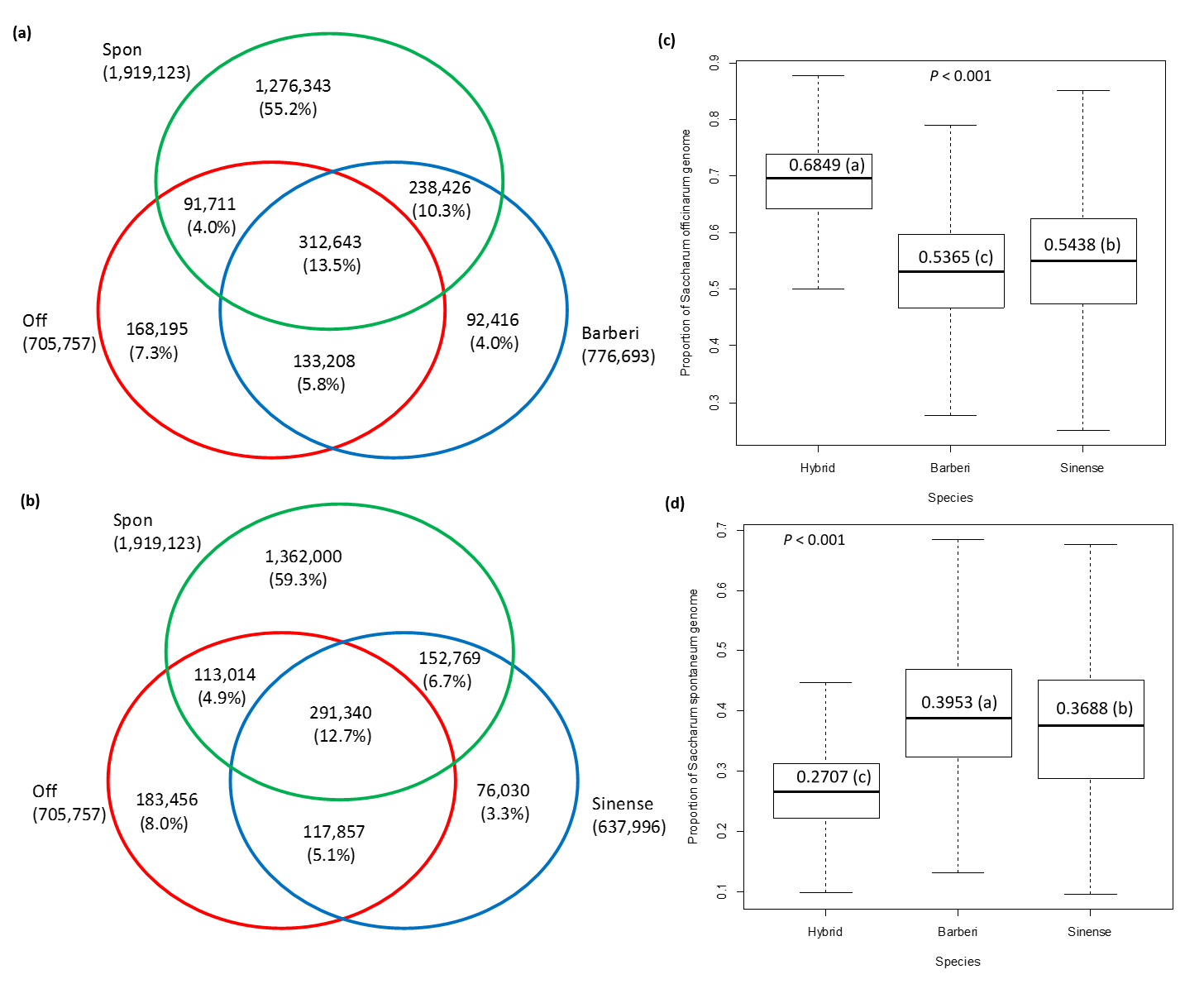


**Figure S4.** Linkage disequilibrium (LD) determined by squared correlation coefficient (r^2^) against distance for each chromosome according to the sorghum genome (Figure S4 (a), (b), (c), (d), (e), (f), (g), (h), (i) and (j) in *S. spontaneum* (green), *S*. *officinarum* (red), and modern *S*. hybrid (blue). Spon = *S. spontaneum*; Off = *S*. *officinarum*; Hybrid = modern *S*. hybrids.


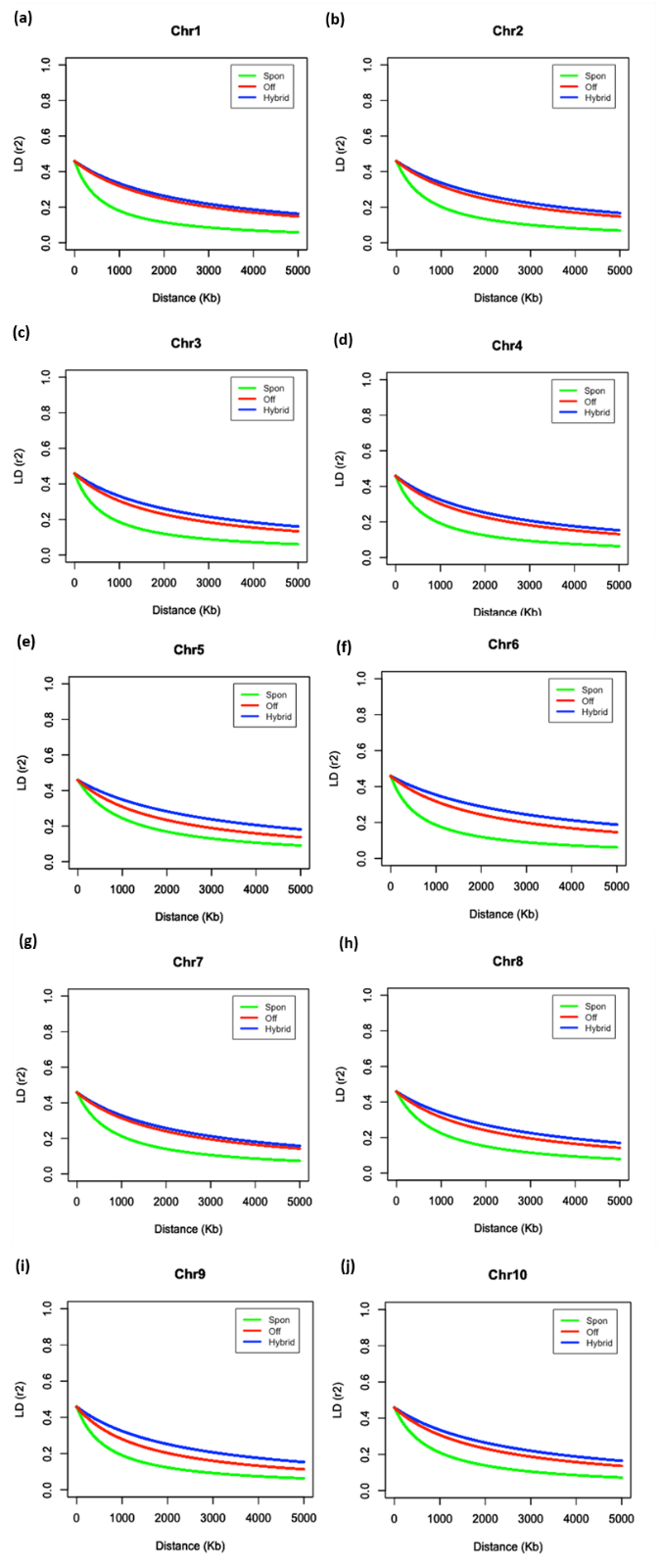


**Figure S5.** Number of domestication and selection genes identified in this study Venndiagram of domestication genes and candidate genes in selective sweep intervals identified for the ancestor of *Saccharum*, *S*. *spontaneum* and *S*. *officinarum* branch respectively (a); domestication genes and selection genes identified from *S. spontaneum* and modern *S*. hybrids, and *S*. *officinarum* and modern *S*. hybrids comparisons (b); Selection genes and candidate genes in selective sweep intervals identified for the ancestor of *Saccharum*, *S*. *spontaneum* and *S*. *officinarum* branch respectively (c). Dom = domestication genes; Spon-Hyb = selection genes identified by *S. spontaneum* and modern *S*. hybrids comparison; Off-Hyb = selection genes identified *S. officinarum* and modern *S*. hybrids comparison; Sacc = candidate genes identified by genome-wide scan in the ancestor of *Saccharum* ; Spon *=* candidate genes identified by genome-wide scan in *S*. *spontaneum*; Off *=* candidate genes identified by genome-wide scan in *S*. *officinarum*; Sel = selection genes identified by *S. spontaneum* and modern *S*. hybrids comparison, and  *S. officinarum* and modern *S*. hybrids comparison.


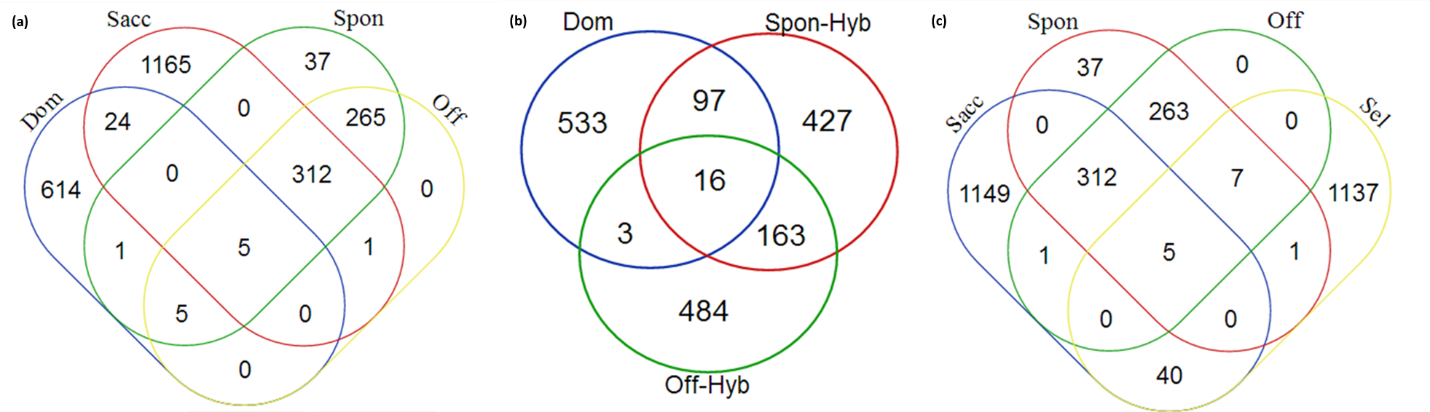


**Figure S6.** Allele frequencies of alternative allele at each SNP locus in gene *Sobic.002G275100*. Non_Sacc = Non-*Saccahrum*; Spon = *S. spontaneum*; Off = *S*. *officinarum*.


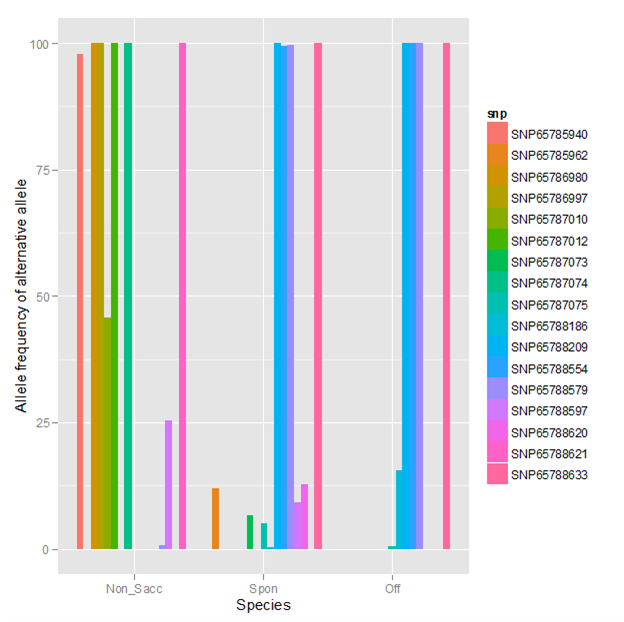


**Figure S7.** Summary of environmental association analyses (EAA). (a) a heat map for pair-wise correlation coefficients among the 104 environmental variables; (b) a plot showed principal components and their corresponding percentages of explained variables; (c), (d), (e) and (f) represented Manhattan plots for PC1, PC2, PC3 and PC4 after EAA, respectively.


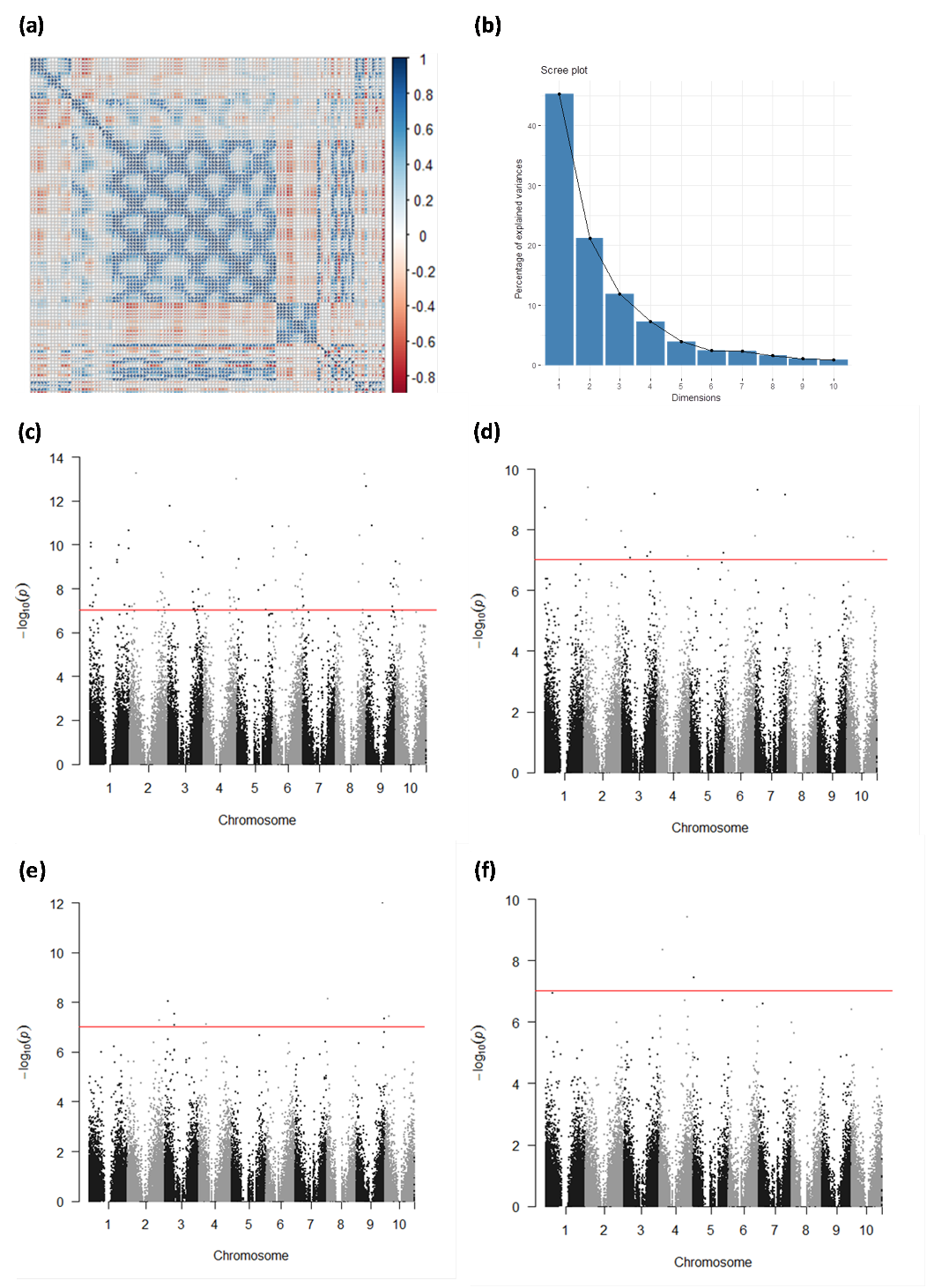

Supplement: Supplementary file 1 — Figure S1 An overview of the sugarcane diversity panel. Figure S2 Ancestry coefficient bar plots for an assumed number of sub‐populations (K) from three to eight. Figure S3 Statistics of species‐specific SNPs in ancient hybrids, S. barberi, S. sinense and modern S. hybrids. Figure S4 Linkage disequilibrium (LD) determined by squared correlation coefficient (r 2) against distance for each chromosome according to the sorghum genome (a), (b), (c), (d), (e), (f), (g), (h), (i) and (j) in S. spontaneum (green), S. officinarum (red) and modern S. hybrid (blue). Figure S5 Number of domestication and selection genes identified in this study Venn diagram of domestication genes and candidate genes in selective sweep intervals identified for the ancestor of Saccharum, S. spontaneum and S. officinarum branch respectively (a); domestication genes and selection genes identified from S. spontaneum and modern S. hybrids, and S. officinarum and modern S. hybrids comparisons (b); Selection genes and candidate genes in selective sweep intervals identified for the ancestor of Saccharum, S. spontaneum and S. officinarum branch respectively (c). Figure S6 Allele frequencies of alternative allele at each SNP locus in gene Sobic.002G275100. Figure S7 Summary of environmental association analyses (EAA). [file PBI-17-488-s003.docx]
